# cGAS/STING/TBK1/IRF3 Signaling Pathway Activates BMDCs Maturation Following *Mycobacterium bovis* Infection

**DOI:** 10.3390/ijms20040895

**Published:** 2019-02-19

**Authors:** Qiang Li, Chunfa Liu, Ruichao Yue, Saeed El-Ashram, Jie Wang, Xiaoli He, Deming Zhao, Xiangmei Zhou, Lihua Xu

**Affiliations:** 1 College of Agriculture, Ningxia University, Xixia District, Yinchuan 750021, China; liqiang5973@163.com (Q.L.); m18295180259@163.com (X.H.); 2 State Key Lab of Agrobiotechnology, National Animal Transmissible Spongiform Encephalopathy Laboratory, College of Veterinary Medicine, China Agricultural University, Beijing 100193, China; 15166486220@163.com (C.L.); yueruichao88@126.com (R.Y.); 18611491892@163.com (J.W.); zhaodm@cau.edu.cn (D.Z.); 3 College of Life Science and Engineering, Foshan University, 18 Jiangwan street, Foshan 528231, China; saeed_elashram@yahoo.com

**Keywords:** *Mycobacterium bovis*, cGAS pathway, dendritic cells, type I interferons, CD4^+^ T cells

## Abstract

Cyclic GMP-AMP synthase (cGAS) is an important cytosolic DNA sensor that plays a crucial role in triggering STING-dependent signal and inducing type I interferons (IFNs). cGAS is important for intracellular bacterial recognition and innate immune responses. However, the regulating effect of the cGAS pathway for bone marrow-derived dendritic cells (BMDCs) during *Mycobacterium bovis* (*M. bovis*) infection is still unknown. We hypothesized that the maturation and activation of BMDCs were modulated by the cGAS/STING/TBK1/IRF3 signaling pathway. In this study, we found that *M. bovis* promoted phenotypic maturation and functional activation of BMDCs via the cGAS signaling pathway, with the type I IFN and its receptor (IFNAR) contributing. Additionally, we showed that the type I IFN pathway promoted CD4^+^ T cells’ proliferation with BMDC during *M. bovis* infection. Meanwhile, the related cytokines increased the expression involved in this signaling pathway. These data highlight the mechanism of the cGAS and type I IFN pathway in regulating the maturation and activation of BMDCs, emphasizing the important role of this signaling pathway and BMDCs against *M. bovis*. This study provides new insight into the interaction between cGAS and dendritic cells (DCs), which could be considered in the development of new drugs and vaccines against tuberculosis.

## 1. Introduction

*Mycobacterium bovis*, the causative agent of tuberculosis in cattle, is an important intracellular pathogen that can cross species barriers and trigger disease in humans and other mammals [1]. *M. bovis* has caused great economic loss and severely threatened public health on a global scale. As a classic intracellular bacterium, *M. bovis* usually causes persistent infection, which triggers disease in humans by direct contact with infected cattle, unpasteurized dairy products, or undercooked meat [2]. In the United States and Mexico, accumulating evidence confirms that those with pulmonary infection and children and persons with HIV co-infection have twice the risk of death from *M. bovis* [3]. In addition, Scott et al. indicated that the burden of *M. bovis* was still underestimated around the world [4].

In recent reports, cyclic GMP-AMP synthase (cGAS) has been identified as a key cytosolic DNA sensor and participant in many innate immune responses [5], particularly in antiviral, anti-infection, and immunologic adjuvants [6,7,8]. The cGAS pathway can be activated by cytosolic pathogenic DNA or self-DNA, which ultimately induces type I interferon (IFN) production. cGAS has also been identified as an important interferon-stimulated gene (ISG) [9]. STING (also referred to as TMEM173, MITA, MPYS, or ERIS) is located downstream of cGAS, which is a critical central adaptor protein and participates in many intracellular signaling pathways, such as IFI16 and DDX41, which are also STING-dependent signaling [10]. Wasserman et al. found that punctate cGAS staining was up-regulated in macrophages during *M. tuberculosis* infection [11], and DNA transfection was co-localized with cGAS in many domains. 

Dendritic cells (DCs), the most potent antigen-presenting cells, initiate and modulate host immune responses via various signaling pathways [12]. Several studies have shown that macrophages were the primary site for *Mycobacterium tuberculosis* (*M. tuberculosis*) infection, but DCs can also be infected [13]. Infected DCs generally enhance the expression of some adhesion molecules and T-cell co-stimulatory molecules, such as CD40, CD80, CD83, and CD86. Generally, these surface markers are highly expressed on mature DCs, which play an important role in antigen presentation and adaptive immune responses.

For *M. tuberculosis* infection, some proteins have been reported to induce the maturation and activation of DCs [14]. In contrast, some research showed that *M. tuberculosis* inhibited DCs maturation and impaired antigen presenting capability [15]. Previous studies usually focused on *M. tuberculosis* with macrophage. However, the mechanism of *M. bovis* infects DCs is still unclear, and the regulatory mechanism of the cGAS signaling pathway in DCs also needs to be investigated. We hypothesized that the maturation and activation of bone marrow-derived dendritic cells (BMDCs) were modulated by the cGAS/STING/TBK1/IRF3 signaling pathway during *M. bovis* infection. Using a targeted knockdown technique, we found that the cGAS pathway was activated by *M. bovis*, which promoted the maturation and antigen presentation of BMDCs. Moreover, our research showed that cGAS and DCs played a critical role in connecting the innate and adaptive immune responses, which would be considered as the particular target for the development of drugs and vaccines against tuberculosis infection.

## 2. Results

### 2.1. cGAS Pathway Is Activated in BMDCs During Mycobacterium bovis Infection

**Figure 1:**
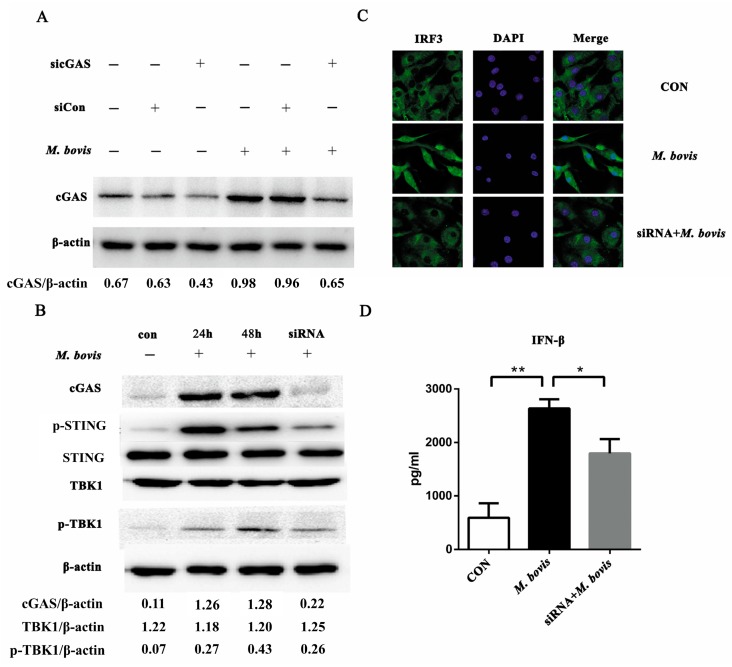
The cyclic GMP-AMP synthase (cGAS) pathway is activated in bone marrow-derived dendritic cells (BMDCs) during *Mycobacterium bovis* infection. (**A**) BMDCs were treated with siRNA, siCon, and *M. bovis*, and cGAS protein was analyzed by Western blotting at 24 h after treatment. (**B**) The related proteins in the cGAS pathway in BMDCs were assayed by Western blotting. The protein levels of cGAS, p-STING, STING, TBK1, and p-TBK1 were analyzed in BMDCs transfected with siCon or sicGAS and then infected for 24 or 48 h with *M. bovis* (MOI 5). (**C**) The co-localization of IRF3 within the nucleus was detected by immunofluorescence microscopy (400 ×). (**D**) The culture supernatants were harvested after 24 h and assessed by ELISA. All data are expressed as mean ± SD, (* *p* < 0.05; ** *p* < 0.01; n.s.: no statistical significance). IFN: interferon.

To determine whether the cGAS pathway could be activated in BMDCs during *M. bovis* infection, we treated BMDCs with siRNA and non-targeting control small interfering RNA (siCon) (Figure 1A, Figures S1 and S2). There were no significant differences with or without siCon treatment, which were identified as the control in subsequent experiments. After *M. bovis* infection, cGAS increased markedly, and its expression was significantly inhibited by cGAS-specific siRNA. For the downstream signaling proteins, p-STING was observably upregulated during infection. There were no significant differences in TBK1 expression among the groups. We further measured phosphorylated-TBK1 (p-TBK1) to determine whether TBK1 was activated. Post-infection 24 and 48 h, p-TBK1 was observably increased, whereas it was decreased in the siRNA group (Figure 1B and Figure S3). Compared to control and siRNA groups, more IRF3 migrated into the nucleus during infection (Figure 1C). Moreover, the production of IFN-β was significantly increased after *M. bovis* infection, whereas it was reduced in the siRNA group (Figure 1D).

### 2.2. cGAS Pathway Promotes Maturation and Activation of BMDCs

**Figure 2:**
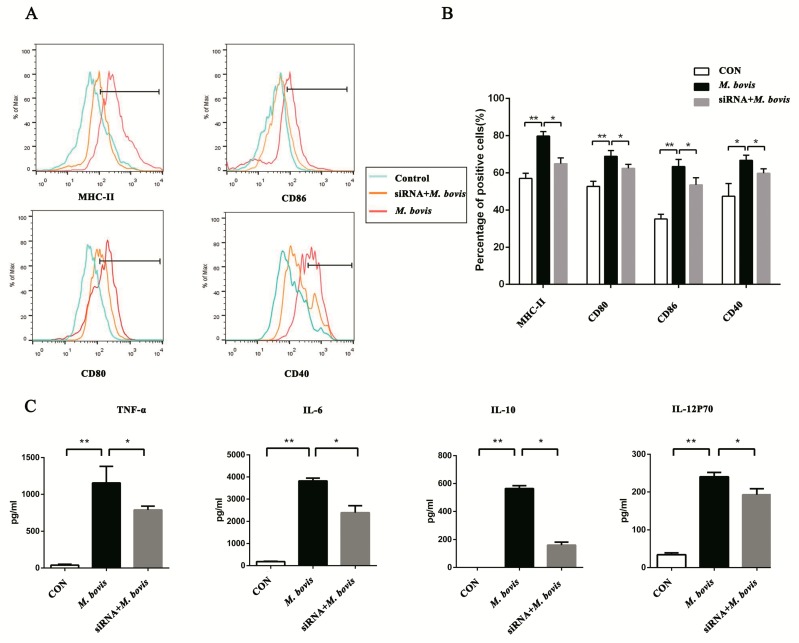
The cGAS pathway promotes the maturation and activation of BMDCs. (**A**) The cell surface markers of CD40, CD80, CD86, and MHC class II were analyzed by flow cytometry in BMDCs transfected with siCon or sicGAS and then infected for 24 h with *M. bovis* (MOI 5). The CD11c marker was used to set the gate for flow cytometric analysis. (**B**) The positive cell rate of surface markers in each group was calculated, and histograms were generated by FlowJo software. (**C**) Culture supernatants were collected after 24 h; the expressions of TNF-α, IL-6, IL-10, and IL-12p70 were assayed by ELISA. All data are expressed as mean ± SD, (* *p* < 0.05; ** *p* < 0.01; n.s.: no statistical significance).

To determine whether the cGAS pathway affects BMDCs maturation and activation after *M. bovis* infection, we assayed the surface markers on BMDCs by flow cytometry (FCM) (Figure 2A). The infection group showed a notable increase in the expression of CD40, CD80, CD86, and MHC class II. However, these were significantly reduced compared to the infected group in the siRNA group (Figure 2B). Further, inflammatory cytokines TNF-α, IL-6, IL-10, and IL-12p70 were detected (Figure 2C). At resting state, the production was undetectable or at low level. Following *M. bovis* stimulation, the expression was observably increased. However, the cytokine secretions were significantly down-regulated in the siRNA group.

### 2.3. Type I Interferon and Its Receptor Contribute to the cGAS Pathway in BMDCs

**Figure 3:**
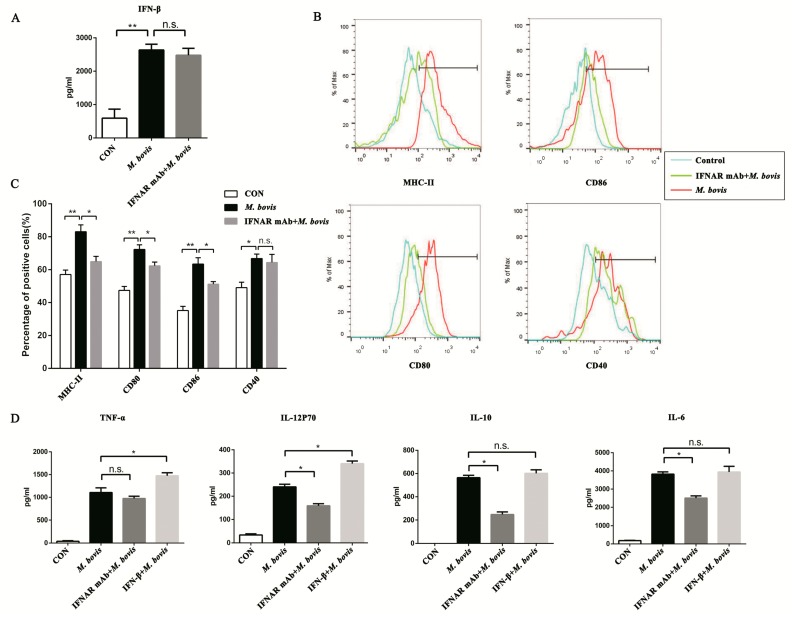
Type I interferon (IFN) and its receptor (IFNAR) contribute to the cGAS pathway in BMDCs. (**A**) The expression of IFN-β was assayed after anti-mouse IFNAR monoclonal antibody (mAb; 10 ng/mL) treatment. (**B**) The cell surface markers of CD40, CD80, CD86, and MHC class II were analyzed by flow cytometry in BMDCs transfected with siCon or sicGAS and then infected for 24 h with *M. bovis* (MOI 5). The CD11c marker was used to set the gate for flow cytometric analysis. The interferon receptor was treated with neutralizing anti-mouse IFNAR mAb (10 ng/mL) to block and then infected for 24 h with *M. bovis* (MOI 5). (**C**) The positive cell rate of surface markers in each group was calculated, and histograms were generated by FlowJo software. (**D**) Culture supernatants were harvested after 24 h; the expression of TNF-α, IL-6, IL-10, and IL-12p70 was assayed by ELISA. IFN-β + *M. bovis*: BMDCs were treated with exogenous IFN-β (10 ng/mL) and then infected with *M. bovis* [16]. All data are expressed as mean ± SD, (* *p* < 0.05; ** *p* < 0.01; n.s.: no statistical significance).

In previous experiments, our results showed that the cGAS signaling pathway could induce IFN-β expression in BMDCs during *M. bovis* infection. We hypothesized that type I IFN would be the key point in the cGAS pathway for BMDCs. In addition, the IFN-β production was not influenced by monoclonal antibodies (mAbs) that block the type I IFN receptor (Figure 3A). In the blocked group (Figure 3B), BMDC surface markers CD80, CD86, and MHC class II were significantly reduced in comparison with the infection group (Figure 3C). Furthermore, the type I IFN receptor (IFNAR) mAb significantly reduced the expression of IL-12p70, IL-10, and IL-6, but TNF-α was not influenced. Interestingly, adding the exogenous IFN-β enhanced the production of TNF-α and IL-12p70; however, IL-10 and IL-6 were not affected (Figure 3D).

### 2.4. BMDCs Promote T cell Activity in the Presence of Type I Interferons

**Figure 4:**
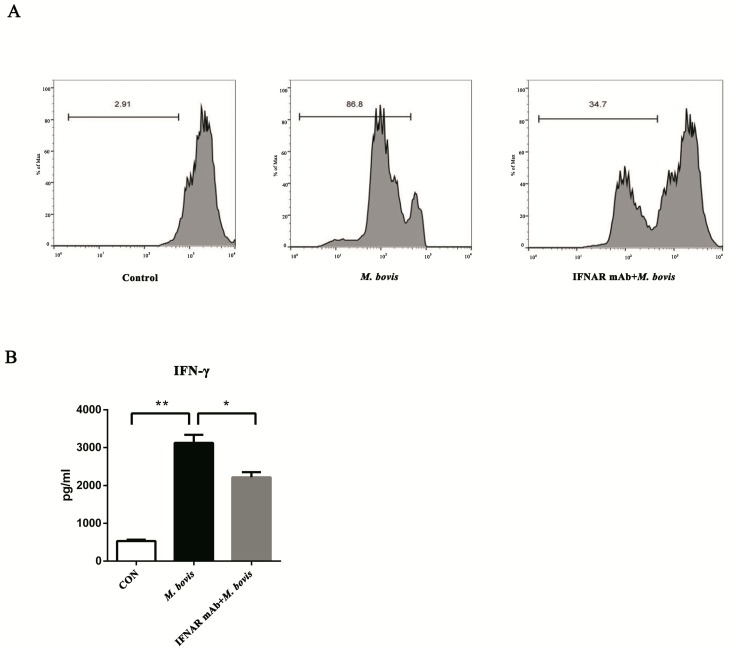
BMDCs promote T cell activity in the presence of type I interferons. (**A**) The proliferation of CD4^+^ T cells was analyzed by flow cytometry after 72 h co-culture. The interferon receptor was treated with neutralizing anti-mouse IFNAR mAb (10 ng/mL) to block and then infected for 24 h with *M. bovis* (MOI 5). CFSE-stained CD4^+^ T cells were co-cultured with three different BMDC groups, and the ratios of BMDC:T cells were 1:10 for 72 h. (**B**) After 72 h co-culture, the culture supernatants were harvested and assessed by ELISA. All data are expressed as mean ± SD. The number above the horizontal bar represents the proliferation rate of CD4^+^ T cell, (* *p* < 0.05; ** *p* < 0.01; n.s.: no statistical significance).

The mature DCs generally present antigens to T cells and induce a cellular immune response. Therefore, we assessed whether type I IFN boosted BMDCs to activate T cell proliferation. The CD4^+^ T cells were labeled with CFSE stain before co-incubation with different treated BMDCs. As expected, the control group displayed nearly no proliferation, it was significantly increased in the single infection group, whereas the proliferation was dramatically inhibited in the blocked group (Figure 4A). Furthermore, the variation trend of IFN-γ production was consistent with T cell proliferation (Figure 4B).

## 3. Discussion

In this study, we identified cGAS as an important signaling pathway that responded to *M. bovis* infection in murine BMDCs. We found that the cGAS pathway was activated and promoted the expression of related signaling proteins during infection. Our data suggested that cGAS recognizing *M. bovis* was a crucial interaction to mediate host immune response, while it also promoted the maturation and activation of BMDCs and up-regulated the expression of some correlative cytokines, such as IL6, IFN-β, and TNF-α. Furthermore, we confirmed that type I IFN and its receptor played an important role in the underlying mechanism to regulate the maturation and activation of BMDCs. Moreover, we found that BMDCs treated with an IFNAR blocking agent inhibited T cell proliferation. 

DCs are potent antigen-presenting cells in the host, linking innate and adaptive immunity. Mature DCs have a strong ability to present antigens to T cells. Some reports found that *M. tuberculosis* triggered maturation and innate immune response in DCs [17], and mediated Th0 differentiation toward the Th1 or Th2 subtypes [18]. However, Satchidanandam et al. suggested that some *M. tuberculosis* proteins inhibited DCs maturation and downregulated the proliferation of T cells [15]. Our data showed that *M. bovis* infection upregulated BMDC surface markers and enhanced T cell proliferation, suggesting that *M. bovis* induces the maturation of BMDCs. Furthermore, cGAS played a crucial role in this process. cGAS is an important intracellular DNA sensor that has been shown to participate in multiple innate immune responses [19,20,21,22,23,24]. Our research showed that the cGAS signaling pathway could be activated in BMDCs during *M. bovis* infection in vitro, and enhanced the type I IFN expression, whereas the cGAS and IFN-β were inhibited in the siRNA group. These results are consistent with previous findings. For example, cGAS was upregulated within *M. tuberculosis*-infected cells, as indicated by punctate cytoplasmic staining [25]. In addition, Teles et al. showed that cGAS^−/−^ mice were more sensitive to *M. tuberculosis* infection [26]. For a different population of DCs, Radtke AJ et al. found that lymph-node resident dendritic cells could capture antigens and elicit CD8^+^ T cell responses [27]. Human plasmacytoid dendritic cells induce type I interferon response by the cGAS-STING signaling pathway [28]. STING showed two bands after *M. bovis* infection, indicating it was activated. Within this signaling pathway, STING recruited TBK-1, which is generally located in the cytoplasm, and showed no significant difference between groups in the present study. Active TBK1 was revealed with a phosphor-specific antibody. IRF3 is a critical interferon regulatory factor, which enters into the nucleus and drives IFN-β transcription after infection. In animal experiments, Manzanillo et al. reported the impairment of IFN-β production during *M. tuberculosis* infection in IRF-3-deficient mice [29].

**Figure 5:**
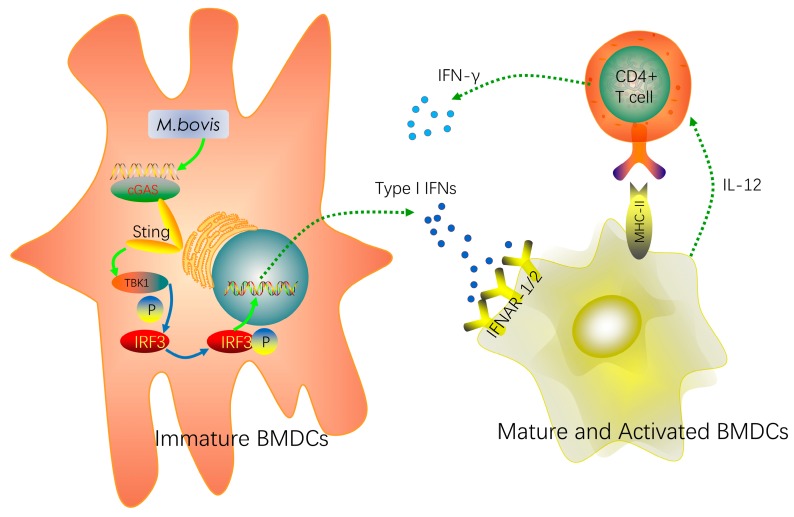
Mediation of the maturation and activation of BMDCs during *Mycobacterium bovis* infection by the cGAS pathway. *Mycobacterium bovis* regulated the maturation and activation of BMDCs via the cGAS/STING/TBK1/IRF3-dependent pathway and promoted type I IFN production. Furthermore, type I IFN and its receptor IFNAR contributed to this process. Moreover, the mature and activated BMDCs enhanced the proliferation of T cells by some surface markers and cytokines. These signaling pathways linked the innate and adaptive immune responses. (The full line arrows signify promotion; the dotted arrows signify releasing cytokines.)

Type I IFN plays an important role in antiviral immunity and intracellular bacteria elimination. IFNAR has a crucial role in inducing adaptive immunity, particularly cytotoxic and CD4^+^ T cell responses. In the IFNAR blocked-infected group, our research showed a significant decrease in the related surface molecules during *M. bovis* infection. However, CD40 was the exception to this effect. It is possible that the CD40 costimulatory protein is not regulated by the type I IFN pathway. This protein was encoded by a member of the TNF-receptor superfamily, but our results suggested that TNF-α was not influenced by the type I IFN pathway. It is possible that its receptor was regulated by the STING-IKK-NF-κB pathway, thus the CD40 marker was not affected. Furthermore, the CD4^+^ T cell proliferation significantly declined in the blocked group. In other words, we found that type I IFN and IFNAR promoted the proliferation of CD4^+^ T cells. Similarly, Hansen et al. confirmed that type I IFN had multiple effects on DCs by several pathways, and was involved in its differentiation, maturation, and migration [30]. The reduction of co-stimulatory marker expression has been reported by Montoya et al. in IFNAR-deficient BMDCs [31]. However, Stanley et al. reported that IFNAR^−/−^ mice were more resistant than wild-type mice to *Francisella. novicida* and *M. tuberculosis* infection, which suggested that type I IFN promoted pathogen survival [32]. These discrepancies may be due to variant cells or pathogen types, and other complicated regulatory networks, which need further research. Ma et al. showed that cGAS expression was specifically regulated by IFNAR signaling, and cGAS expression was induced by type I IFN feedback regulation [33]. Bode et al. reported that the cGAS-STING pathway-dependent type I IFN expression in DCs may be beneficial for patients who have autoimmune diseases, cancer, or infectious diseases [28]. Recently, type I IFNs have been used to treat chronic viral hepatitis, certain cancers, and multiple sclerosis [34]. Most importantly, our results suggest that type I IFN expression in BMDCs might act in an autocrine or paracrine positive feedback regulatory mechanism via the cGAS signaling pathway, strengthening the host immune response (Figure 5).

Diverse cytokines are essential for host immune responses against infection or tissue injury. TNF-α, IL-12p70, IL-10, and IL-6 were secreted by BMDCs to respond to *M. bovis* infection. Interestingly, TNF-α declined during the period that cGAS was treated with siRNA. However, it was not influenced after IFNAR blockage, which suggested that TNF-α secretion is independent of the type I IFN signaling pathway. Abe et al. [35] found that STING could mediate the activation of NF-κB/IRF3 and promote pro-inflammatory gene transcription. Their findings suggested that cGAS regulated TNF-α indirectly via the STING-IKK-NF-κB pathway, and therefore TNF-α production was not influenced by the IFNAR pathway. Lienard et al. [36] also confirmed that IL-10 and IL-6 were significantly decreased after knocking out IFNAR, whereas TNF-α was not changed. These findings are highly consistent with our results. IL-10 is a major anti-inflammatory cytokine, and it participates in multiple regulations. Ersoy et al. [37] found that when multiple sclerosis patients were treated with IFN-β, the IL-10 levels were significantly increased. In murine experiments, IFN-β could promote antigen-specific T cells to express IL-10 [38]. On the contrary, Feng et al. [39] found that IFN-α and IFN-β prevented the production of IL-10 at the mRNA and protein levels in activated monocytes. Our findings suggested that type I IFN promoted the expression of some inflammatory cytokines and induced a host immune response. In the review of tuberculosis pathogenesis, Etna et al. [40] indicated that the pro and anti-inflammatory cytokines are a double-edged sword, balancing immune protection and evasion during infection.

In conclusion, we provide evidence that the cGAS signaling pathway was activated during *M. bovis* infection and mediated the maturation and activation of BMDCs. Furthermore, we reveal the potential regulatory mechanism of type I IFN and its receptor for the cGAS pathway. Moreover, we highlight the role of BMDCs between innate and adaptive immunity. Our research provides new insights into the cGAS pathway, DCs, and type I IFN, which could be considered to develop prevention or immunotherapy strategies against tuberculosis.

## 4. Materials and Methods

### 4.1. Ethics Statement

All protocols and procedures were performed according to the Chinese Regulations of Laboratory Animals—The Guidelines for the Care of Laboratory Animals (Ministry of Science and Technology of People’s Republic of China) and Laboratory Animal Requirements of Environment and Housing Facilities (GB 14925–2010, National Laboratory Animal Standardization Technical Committee). The license number associated with the research protocol was 20110611-01. The animal study proposal was approved by the Laboratory Animal Ethical Committee of China Agricultural University (approved date: 11 June 2011).

### 4.2. Mice

Six- to eight-week-old female C57BL/6 mice were purchased from Beijing Vital River Laboratory Animal Technology Co., Ltd (Beijing, China). Mice were exposed to a 12 h/12 h light/dark regimen at 25 °C. All mice were maintained under specific pathogen-free conditions, and all efforts were made to minimize mouse suffering.

### 4.3. Cell Preparation

BMDCs were prepared using a previously described method [41]. Femurs and tibias of 6–8 week-old female mice were separated and surrounding muscle tissue was removed. Then, intact bone was put in 75% ethanol for 2–5 min and washed with PBS. This was cut at both ends and marrow was flushed with PBS by syringe. After being washed, primary cells were cultured in bacteriological Petri dishes with 100 mm diameter. Cell culture medium was RPMI-1640 (GIBCO BRL, Eggenstein, Germany) supplemented with streptomycin (100 μg/mL, Sigma, St. Louis, MO, USA), l-glutamine (2 mM, Sigma), 10% fetal calf serum (GIBCO BRL), and 10 ng/mL recombinant murine GM-CSF (315-03, PeproTech, Rocky Hill, NJ, USA). After 7 days, cells were harvested and analyzed by flow cytometry (FCM). 

### 4.4. siRNA Transfection

BMDCs were cultivated in a 12-well plate. Subsequently, the cell culture medium was replaced, and cells were transfected with 20 ng/mL non-targeting control small interfering RNA (siCon) and cGAS-specific siRNA (L-055608-01-0005, GE Dharmacon, Fairfield, CT, USA) using transfection reagent (Polyplus Transfection, Illkirch, France) according to the manufacturer’s instructions. After 48 h of transfection, the efficiency of the knockdown was confirmed by Western blotting.

### 4.5. M. bovis Infection

The *M. bovis* were cultured in 7H9 Middlebrook media (BD Biosciences, New York, NY, USA) containing albumin-dextrose-catalase (ADC) enrichment solution with 0.05% Tween-80 (Difco, New York, NY, USA), and grown to mid-logarithmic phase for 1 week at 37 °C.

Experimental groups were as follows: control group, non-stimulated BMDCs throughout the whole process; *M. bovis* group, BMDCs only infected with *M. bovis* (CFU/Cell = 5:1); siRNA + *M. bovis* group, BMDCs were pre-treated with siRNA and then infected with *M. bovis*; IFNAR mAb + *M. bovis* group, BMDCs were pre-treated with the neutralizing anti-mouse IFNAR mAb (10 ng/mL, MAR1-5A3, Biolegend, San Diego, CA, USA) to block the IFNAR and then infected with *M. bovis*; IFN-β + *M. bovis* group, BMDCs were pre-treated with exogenous IFN-β (10 ng/mL, ab24324, Abcam, Cambridge, UK) and then infected with *M. bovis.*

### 4.6. Western Blotting

Western blotting was performed as previously described [42]. Briefly, cells were lysed in cold radio immunoprecipitation assay (RIPA) lysis buffer for 30 min and centrifuged to remove precipitates. Loading buffer was added, then samples were boiled for 10 min. Proteins were separated by SDS-PAGE and transferred to PVDF membranes, which were washed with Tris-buffered saline plus Tween-20 (TBST) and blocked with 5% non-fat milk. Next, the membranes were incubated with primary antibodies overnight at 4 °C, washed with TBST, then incubated with horseradish peroxidase (HRP)-labeled secondary antibodies. Finally, an enhanced chemiluminescence system was used to detect proteins according to the manufacturer’s protocol. The antibodies used were rabbit anti-cGAS (D3080, CST, anti-MB21D1, Boston, MA, USA), rabbit anti-STING (19851-1-AP, Proteintech, Chicago, IL, USA), rabbit anti-TBK1 (ab40676, Abcam, Cambridge, UK), and p-TBK1 (ab109272, Abcam).

### 4.7. Enzyme-Linked Immunosorbent Assay

Supernatants were collected for analysis of IFN-β (CSB-E04945m), IL-12p70 (KE10014), IL-10 (KE10008), IL-6 (KE10007), TNF-α (KE10002), and IFN-γ (CSB-E04578m) using the mouse enzyme-linked immunosorbent assay (ELISA) kits (Proteintech and Cusabio, Chicago, IL, USA). All processing was strictly according to the manufacturer’s instructions.

### 4.8. Flow Cytometry

Cells were harvested and washed with PBS, and subsequently exposed to mouse antibody against MHC class II (11-5322-81), CD80 (11-0801-81), CD86 (11-0862-81), and CD40 (11-0402-81), as well as anti-CD11c mAb (17-0114-82) at room temperature 30 min away from light (eBioscience, San Diego, CA, USA). Then, cells were washed three times in PBS and re-suspended in the fixation buffer. Samples were detected by an LSR II (BD Biosciences) flow cytometer and analyzed with FlowJo software (TreeStar, Ashland, OR, USA). All operations were per manufacturer’s instructions.

### 4.9. Proliferation of CD4^+^ T Cells

CD4^+^ T cells were isolated from the spleens of female C57BL/6 mice (4–6 weeks old) by magnetic-activated cell sorting (MACS) columns (130-104-454, Miltenyi Biotec, Bergisch Gladbach, Germany). Then, CD4^+^ T cells were stained with 1 μM carboxyfluorescein (CFSE, C34554, Invitrogen, Carlsbad, CA, USA), as previously described [43]. Following BMDC infection with *M. bovis*, CFSE-stained CD4^+^ T cells were co-cultured with BMDCs in different groups at a BMDC:T cell ratio of 1:10. After 3 days of co-culture, cells were collected for flow cytometry analysis, and culture supernatants were collected for cytokine assays.

### 4.10. Statistical Analysis

All experiments were repeated at least three times with consistent results. Data were expressed as the mean value ± SD. The differences were evaluated by ANOVA test using the 95% confidence level in Tukey’s multiple comparisons. All data were calculated and analyzed with SPSS 22.0 software, and the graphs were generated in GraphPad Prism 7 software (San Diego, CA, USA). The Western blot density was analyzed by ImageJ. Values of *p* < 0.05 were considered statistically significant. Statistical significance was expressed as follows: *, *p* < 0.05; **, *p* < 0.01; ***, *p* < 0.001.

## Supplementary Materials

Supplementary materials can be found at http://www.mdpi.com/1422-0067/20/4/895/s1.
