# Supplementary material for: cGAS/STING/TBK1/IRF3 Signaling Pathway Activates BMDCs Maturation Following Mycobacterium bovis Infection
Source: Int J Mol Sci. 2019 Feb 19;20(4):895. doi: 10.3390/ijms20040895 (PMC6412216; doi:10.3390/ijms20040895)
Supplement: Supplementary file 1 [file ijms-20-00895-s001.pdf]

International Journal of
Molecular Sciences

MDPI

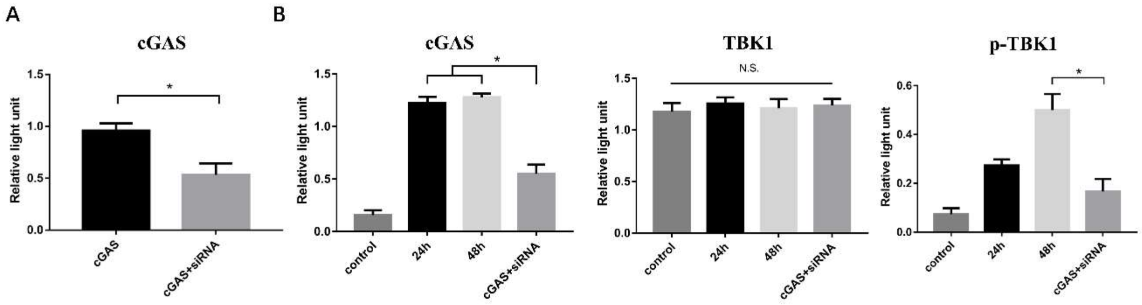

Figure S1. The densitometric analysis of Western blots. (**A**) In the absence of infection，BMDCs were treated with siRNA, and cGAS protein was analyzed by Western blotting at 24 h after treatment. (**B**) The cGAS, TBK1, and p-TBK1 proteins of cGAS pathway in BMDCs were analyzed in BMDCs transfected with siCon or sicGAS and then infected for 24 or 48 h with *M. bovis* (MOI 5). All data are expressed as mean ± SD, (* $p < 0.05$; ** $p < 0.01$; n.s.: no statistical significance).

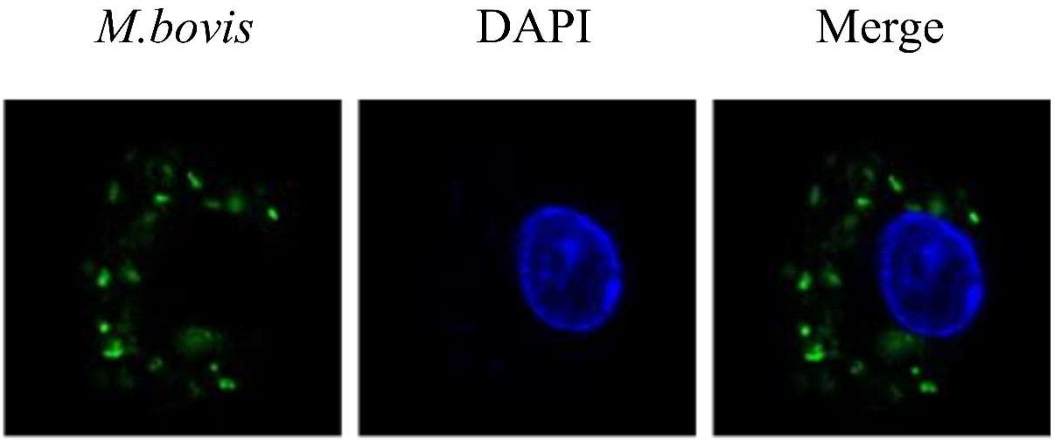

Figure S2. The *M. bovis* infection process of BMDCs. The co-localization of *M. bovis* and cell nucleus was detected by immunofluorescence microscopy in BMDCs.

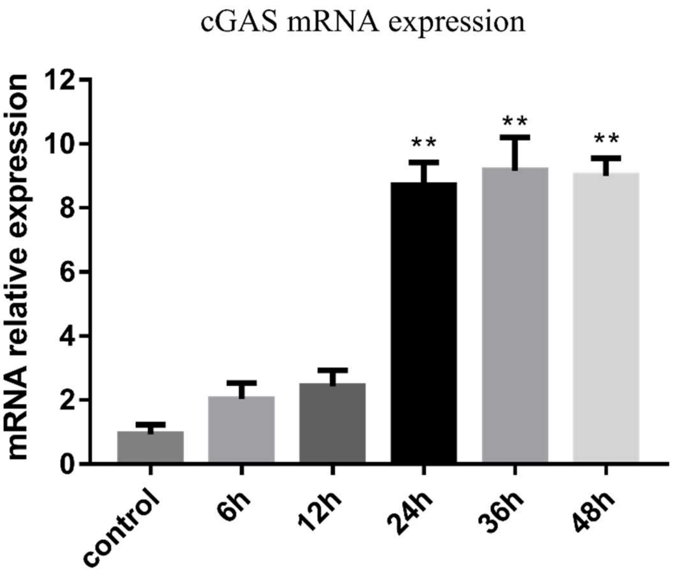

Figure S3. The cGAS mRNA expression of *M. bovis*-infected BMDCs in different time points. The expression of cGAS mRNA was detected by qRT-PCR after 6, 12, 24 , 36, and 48 h of infection. All data are expressed as mean ± SD, (* $p < 0.05$; ** $p < 0.01$; n.s.: no statistical significance).

© 2019 by the authors. Submitted for possible open access publication under the terms and conditions of the Creative Commons Attribution (CC BY) license (http://creativecommons.org/licenses/by/4.0/).
